# Effect of dolomite and biochar addition on N_2_O and CO_2_ emissions from acidic tea field soil

**DOI:** 10.1371/journal.pone.0192235

**Published:** 2018-02-02

**Authors:** Aung Zaw Oo, Shigeto Sudo, Hiroko Akiyama, Khin Thuzar Win, Akira Shibata, Akinori Yamamoto, Tomohito Sano, Yuhei Hirono

**Affiliations:** 1 Institute for Agro-Environmental Science (NIAES), National Agriculture and Food Research Organization (NARO), 3-1-3 Kannondai Tsukuba, Ibaraki, Japan; 2 Central Regional Agricultural Research Center, National Agriculture and Food Research Organization (NARO), Kannondai 2-1-18, Tsukuba, Japan; 3 Ritsumeikan University, Kyoto, Japan; 4 Tokyo Gakugei University, Koganei, Tokyo, Japan; 5 Institute of Fruit Tree and Tea Science, National Agriculture and Food Research Organization (NARO), 2769 Kanaya-Shishidoi, Shimada, Shizuoka, Japan; RMIT University, AUSTRALIA

## Abstract

A laboratory study was conducted to study the effects of liming and different biochar amendments on N_2_O and CO_2_ emissions from acidic tea field soil. The first experiment was done with three different rates of N treatment; N 300 (300 kg N ha^-1^), N 600 (600 kg N ha^-1^) and N 900 (900 kg N ha^-1^) and four different rates of bamboo biochar amendment; 0%, 0.5%, 1% and 2% biochar. The second experiment was done with three different biochars at a rate of 2% (rice husk, sawdust, and bamboo) and a control and lime treatment (dolomite) and control at two moisture levels (50% and 90% water filled pore space (WFPS)). The results showed that dolomite and biochar amendment significantly increased soil pH. However, only biochar amendment showed a significant increase in total carbon (C), C/N (the ratio of total carbon and total nitrogen), and C/IN ratio (the ratio of total carbon and inorganic nitrogen) at the end of incubation. Reduction in soil NO_3_^-^-N concentration was observed under different biochar amendments. Bamboo biochar with the rates of 0.5, 1 and 2% reduced cumulative N_2_O emission by 38%, 48% and 61%, respectively, compare to the control soil in experiment 1. Dolomite and biochar, either alone or combined significantly reduced cumulative N_2_O emission by 4.6% to 32.7% in experiment 2. Reduction in N_2_O production under biochar amendment was due to increases in soil pH and decreases in the magnitude of mineral-N in soil. Although, both dolomite and biochar increased cumulative CO_2_ emission, only biochar amendment had a significant effect. The present study suggests that application of dolomite and biochar to acidic tea field soil can mitigate N_2_O emissions.

## Introduction

Nitrous oxide (N_2_O) is a potent greenhouse gas and the single most important ozone depleting compound currently emitted to the atmosphere [1]. Agricultural soil is the single largest source of global anthropogenic N_2_O emission [2] due to widespread use of synthetic nitrogen (N) fertilizer. Accounting for approximately 59% of anthropogenic emissions [3], agriculture is a sector with a considerable mitigation potential [4]. Reducing agricultural N_2_O emissions would reduce the GHG induced radiative forcing [4] and improve the stability of the stratospheric ozone layer [1]. Therefore, it is urgent to establish effective agricultural management practices that can mitigate GHG emissions.

Tea (*Camellia sinensis*) is cultivated widely in Japan, and the fields are typically located on acidic soil. Tea is a leaf-harvested crop, and nitrogen is the most important nutrient for improving the yield and quality of tea leaves [5]. Hence, to meet these criteria, tea fields in Japan tend to receive higher rates of N fertilizer than other crops, sometimes exceeding 1,000 kg N ha^-1^ year^-1^ [6], resulting in problems such acidification of soil and high rates of N_2_O emissions [5]. Strongly acidic soils have a negative impact on tea production [7] and long-term soil acidity enhances the N_2_O emission potential from soil [5]. Akiyama et al. [6] reported that the mean fertilizer-induced N_2_O emission factor of tea fields is higher than that of other upland fields. Since tea fields account for 16% of the total N_2_O emissions from agricultural soils in Japan [8], reducing N_2_O emissions from tea fields will greatly reduce N_2_O emission from agriculture in Japan.

Liming is a common agricultural practice to counteract soil acidification. It plays an important role in regulation of soil processes such as organic matter mineralization, N transformation, nitrification, and denitrification, which in turn affect soil N_2_O production [9, 10]. Lime application in acidic soil increases C and N mineralization [11], enhances nitrification and denitrification rates [12] and therefore enhances N_2_O emissions. Conversely, others have reported a decrease in N_2_O emission when lime was applied to acidic soils [13, 14]. No single consensus exists on the effect of lime application on N_2_O emissions from acidic soils.

Recently, attention has focused on the effect of biochar amendment on soil gas fluxes. Biochar amendment affects C and N turnover by influencing microbial community structure and biomass [15], and hence alters CO_2_ and N_2_O emissions from soil [16]. The effect of biochar addition on soil CO_2_ and N_2_O fluxes has been extensively investigated, but the results have not been consistent. Biochar application to soil could affect N_2_O emissions by (i) altering soil properties and the availability and distribution of key electron acceptors (O_2_, NO_3_^-^), and donors (NH_4_^+^, dissolved organic matter), (ii) inducing catalytic reduction of N_2_O to N_2_ following oxidation and subsequent reactions of biochar with soil minerals, and (iii) influencing microbial community structures, and microbial enzymes and processes (N mineralization-immobilization turnover, nitrification, denitrification) involved in N cycling in soil [16, 17]. However, the influence of different types of biochar on soil properties could be highly variable [18], because biochar properties vary widely, depending on the biomass source and pyrolysis conditions [19]. Some authors suggested that the elevated soil pH is responsible for reduced N_2_O emissions following biochar application through increased activity of N_2_O reducing bacteria [20]. Biochar addition can also markedly affect soil CO_2_ emission [21]. In the literature, biochar addition has been reported to have positive, negative, or negligible effects on soil CO_2_ efflux [22, 23, 24, 25]. The effect of biochar addition on CO_2_ emission can depend on the time since biochar addition [23], biochar type and application rate, soil type, local environmental condition, and management practices used [21]. To date, no single consensus exists on net impact of biochar amendment on N_2_O and CO_2_ emissions in agricultural soils.

The raw materials of biochar used in agriculture are ranging from waste woods to some flammable substances such as agricultural Bi-products. In this study, three different biochar were used to study the effect of biochar amendment on greenhouse gas emission from acidic tea field soil. Carbonization of bamboo and its utilization has been widely noticed as one of the management methods of bamboo forest, which occupies wide area in Southwest Japan. Rice husk and saw dust were major by-products of rice farming and wood working operation, respectively. There is still limited information on the effect of different biochar carbonized from different wastes on soil pH related N_2_O and CO_2_ emissions from strongly acidic soils.

Therefore, to understand the potential effects of different biochar and liming treatments on N_2_O fluxes from acidic tea field soil, it is necessary to measure N_2_O fluxes at both low and high soil water contents since nitrification may be predominant at low soil moisture content and denitrification at high soil moisture content. The objective of the first experiment was to investigate the effect of different biochar and N application rates on soil N_2_O and CO_2_ emissions from acidic soil. The second experiment was conducted to evaluate the effect of three different biochar and dolomite application on N_2_O and CO_2_ emissions at two water levels.

We hypothesized that (1) bamboo biochar with high rates of amendment showed high reduction in N_2_O emissions by increasing soil pH, (2) N_2_O emissions are also reduced following the application of different biochar to soil, and (3) similar reduction in N_2_O emission was possible when soil pH is increased by dolomite application.

## Materials and methods

### Soil and biochar preparation

The soil used in this study was collected from a tea field at the Institute of Fruit Tree and Tea Science, National Agriculture and Food Research Organization (NARO), Shizuoka, Japan (34° 480′N, 138° 08′E). The soil was classified as Andosol. Soil samples were collected at a depth of 0–10 cm from multiple points of a selected field. Soil samples were mixed to obtain a composite sample for soil analysis and the incubation study. The soil was composed of 14% sand, 43% silt, and 43% clay [26] and other soil physiochemical properties included total N of 6.5 g kg^-1^, total C of 57.7 g kg^-1^, NH_4_^+^-N of 259.1 mg kg^-1^, NO_3_^-^-N of 107.5 mg kg^-1^, pH of 3.08 (1:5 H_2_O), and EC of 748 μS cm^-1^.

Three different biochar types produced from different feedstocks [bamboo biochar (bamboo), rice husk biochar (rice husk), and sawdust biochar (sawdust)] were used. Bamboo (Mosou bamboo and Madake bamboo) feedstocks were harvested from bamboo forest in Hozu district, Kameoka city, Kyoto, Japan. Bamboo culms were chopped to around 1.5 meter. The open burn kiln used in this study was 534 L in capacity with diameter and height of 150 m and 43 cm, respectively (S1 Fig). The chopped bamboo feedstocks were heated in an open burn kiln. The open fire kiln is an “auto-thermal” process and burns part of the feedstock material to heat the rest of the material and turn it into char. Pyrolysis temperature at pyrolysis zone was approximately 500–600°C. Rice husks, major by-products of rice milling were collected by local farmer from Yatabe, Tsukuba city, Ibaraki prefecture, Japan. For charring process, rice husks were pyrolyzed using an open-type carbonizer with attached chimneypiece. Carbonization temperature was approximately 500–560°C. Sawdust biochar was made by the Nara Tanka Factory at a pyrolysis temperature of 800°C. Biochar characteristics are shown in S1 Table.

### Experimental design and incubation study

#### Experiment 1

A 64 days incubation experiment was conducted. A total of 100 g of air-dried soil (2-mm sieve) was added to a polypropylene jar (750 ml). The soil moisture was adjusted to 80% water filled pore space (WFPS) by carefully spraying deionized water on to the soil. Then the soils were pre-incubated at 25°C and 80% WFPS for 8 days in an incubator in the dark, in order to revive soil microbial activity.

The experiment was laid out two-factor completely randomized design with three replications. Three nitrogen fertilizer treatments were used as factor one; (1) 13.34 mg, (2) 26.67 mg and (3) 40 mg N per 100 g dry soil, which were equivalent to 300 kg (N 300), 600 kg (N 600) and 900 kg (N 900) N ha^-1^ application rates, respectively. Ammonium sulfate fertilizer was used as N source. All the N fertilizers were dissolved in water for application. The split application of N fertilizer was done at 0, 16, 32 and 46 days during incubation. Four biochar treatments were used as factor two: (1) 0% (B 0%), (2) 0.5% (B 0.5%), (3) 1% (B 1%) and (4) 2% (B 2%) biochar, which were equivalent to 0, 5, 10 and 20 t ha^-1^ if calculated based on 10 cm incorporation depth in the field. After pre-incubation, biochars were added to soils with thorough mixing. All the treated jars were incubated aerobically at a constant temperature of 25°C in an incubator. During incubation, water loss was monitored periodically by weighing the jars and adjusted to 80% WFPS with the addition of deionized water.

#### Experiment 2

A 60 days incubation experiment was conducted. A total of 75 g of air-dried soil (2-mm sieve) was added to a polypropylene jar (750 ml). The soils were wetted with deionized water to 50% WFPS and pre-incubated for 14 days at 25°C for the activation and stabilization of microbial activities prior to the addition of treatments. After pre-incubation, soil was treated with the following treatments in a split-split plot factorial design. One of the three different types of biochar or control was applied [hereafter, referred to as B0 (control), RH (rice husk), SD (sawdust), and BB (bamboo)] at a rate of 2% by weight which was equivalent to 20 t ha^-1^ if calculated based on 10 cm incorporation depth in the field. Biochar treatments were further split into samples with and without dolomite treatments. Dolomite (CaMg(CO_3_)_2_) was used in this experiment which contained 10% MgCO_3_, 80% CaCO_3_, and 10% other constituents. Dolomite used in this study was produced by Shimizu Sekkai Kogyo Co. Ltd., Yamasugecho, Sano district, Tochigi city, Japan. In a preliminary experiment, the amount of dolomite needed was estimated by a lime requirement test using 0.1N NaOH solution. This was done by monitoring soil pH over a period of 1 day after adding various amount of NaOH. The amount of dolomite needed was 3.5 t ha^-1^ to increase the initial soil pH of acidic tea field soil by 1 unit. Biochar and dolomite treatments were tested at 50% and 90% WFPS. Following pre-incubation, dolomite, biochar, and ammonium sulfate (equivalent to 220 mg N kg^-1^ soil) were added to soils with thorough mixing. All the treated jars were incubated aerobically at a constant temperature of 30°C in an incubator. To prevent moisture loss, aluminum sheets were placed over the top of each jar, and pinholes were pierced to allow gas exchange. The moisture content of the soil was maintained at 50% or 90% WFPS throughout the experiment by weighing the jars twice a week and adding deionized water if needed. Additionally, polyethylene bottles containing 10 g of soil for each treatment were prepared (n = 3) and maintained in the same conditions to measure for soil mineral N extracted on 8 and 21 days of incubation.

### Gas sampling and analysis

During incubation period, gas samples from all the jars were collected 22 times in both experiments. The jars were thoroughly flushed with ambient air and left opened for approximately 30 min to equilibrate with the atmosphere [27]. The jars were then sealed for 30 minutes using lids with a rubber septum for gas sampling. These lids were only used during gas sampling and were replaced with the aluminum sheet for the rest of the experiment. Gas samples were drawn from the incubation jar using a 50-ml syringe. The air inside the jar was thoroughly mixed by flushing the syringe three times before collecting the gas samples. The sample gasses were then transferred to 15-mL vacuum glass vials with rubber stoppers, and kept cool and dark until analysis. The concentrations of N_2_O and CO_2_ were analyzed with an automated analysis system for three gases of CO_2_, CH_4_, and N_2_O. This system consists of two gas chromatographs (GC-14B, Shimadzu, Kyoto, Japan), of which one has both a thermal conductivity detector (TCD) and a flame ionization detector (FID), and the other has an electron capture detector (ECD). This system can analyze 80 samples consecutively with a modified automated headspace sampler (AOC-5000, Shimadzu, Kyoto, Japan). The difference in gas concentrations between the atmosphere and samples was used to calculate N_2_O and CO_2_ fluxes. The cumulative gas emissions from each jar were calculated by summing their respective emissions over total incubation days [27].

### Soil analysis

Soil pH was measured in the supernatant suspension of 1:5 soil: H_2_O solution using a pH meter (Mettler Toledo, FiveEasy, FE 20). Soil mineral N contents (NO_3_^-^ and NH_4_^+^) were determined from 10 g fresh soil extracted with 50 ml 1 M KCl and analyzed using a QuAAtro Auto Analyzer (BLTEC, Tokyo, Japan). Soil total N and total C contents were analyzed by using a NC analyzer (Sumigraph NC-80; Sumika Chemical Analysis Service Co., Japan). Soil C/N ratio was the ratio of total C to total N. Inorganic nitrogen (IN) was the sum of the NH_4_^+^ and NO_3_^-^ concentrations. Soil C/IN ratio was the ratio of total C to IN.

### Statistical analysis

The effect of dolomite, biochar and soil moisture on soil properties and cumulative gas emissions were tested by the analysis of variance (ANOVA) using CropStat 7.2 statistical software program. Principle component analysis was done using XLSTAT 2016 version (Addinsoft).

## Results

### Soil properties

#### Experiment 1

Biochar amendment significantly affected (p<0.05) soil pH after 64 days of incubation (Table 1). The biochar dose-dependent rise in soil pH was pronounced in all N treated soils with the highest value observed in soils treated with 2% biochar (B 2%). Although significant differences in soil pH was observed under biochar amendment, only 0.09 units pH increased was observed in soil treated with N 300 and 0.07 units treated with N 600 and N 900.

**Table 1:** Soil total N, total C, pH, EC, NH4—N and NO3—N content after 64 days of incubation (Experiment 1). Data analyzed by two-way ANOVA with the factors of Nitrogen (N) and Biochar (B). Values are mean ± standard variation.

Nitrogen	Biochar (%)	Total N (g kg^-1^)	Total C (g kg^-1^)	Soil pH	NH_4_^—^N (mg kg^-1^)	NO_3_^-^-N (mg kg^-1^)
N 300	B 0	6.3±0.8	65.6±5.9	3.54±0.03	457.5±42.9	231.7±12.4
	B 0.5	5.8±0.8	69.1±2.7	3.59±0.01	453.9±41.5	222.1±2.5
	B 1	6.2±1.1	77.8±9.6	3.61±0.01	428.1±15.4	215.7±5.6
	B 2	6.2±1.1	84.0±9.6	3.63±0.01	408.9±33.7	192.1±16.9
N 600	B 0	6.0±0.4	64.0±2.6	3.55±0.01	670.3±41.6	238.1±13.1
	B 0.5	5.7±0.1	68.2±3.3	3.59±0.01	640.6±58.8	215.9±13.9
	B 1	5.8±1.5	73.2±10.8	3.60±0.02	590.8±33.4	199.4±12.5
	B 2	6.9±1.1	94.3±9.2	3.63±0.02	558.7±40.5	205.2±5.1
N 900	B 0	6.3±0.6	66.8±8.2	3.56±0.01	763.8±40.6	240.4±15.5
	B 0.5	7.3±1.2	74.6±12.9	3.60±0.01	752.2±36.5	221.5±12.3
	B 1	6.0±0.1	76.6±8.0	3.61±0.01	701.4±49.5	212.7±8.2
	B 2	6.5±0.7	86.0±8.1	3.62±0.01	734.1±34.5	199.9±11.1
Analysis of Variance						
	N	ns	ns	ns	**	ns
	B	ns	**	**	**	**
	N x B	ns	ns	ns	ns	ns

*P<0.05

**P<0.01, ns = not significance at 0.05 level

After 64 days of incubation, soil total N content was not affected by N fertilizer application and biochar amendment (Table 1). Biochar amendment significantly affected (p<0.05) soil total C content. Soil total C content increased with increasing addition of biochar by 7.8%, 15.8% and 34.7% averaged over all N treatments under B 0.5%, B 1% and B 2%, respectively, compared to no amendment. Soil NH_4_^-^-N concentration was significantly affected (p<0.01) by N fertilizer application and biochar amendment but there was no interaction between the two treatments (Table 1). Among the N rates, the highest NH_4_^-^-N concentration (763.9 g kg^-1^ soil) was observed in N 900 followed by N 600 (670.2 g kg^-1^ soil) and the lowest content (457.1 g kg^-1^ soil) with N 300 treatment. Soil NH_4_^-^-N concentration decreased with increasing addition of biochar by 0.2%, 3.1% and 6.7% averaged over all N treatments under B 0.5%, B 1% and B 2%, respectively, compared to no amendment. Soil NO_3_^-^-N concentration was not affected by N fertilizer application after 64 days of incubation. Biochar application significantly affected (p<0.05) NO_3_^-^-N concentration. The NO_3_^-^-N concentration decreased with increasing rate of biochar amendment by 7.1%, 11.5% and 15.9% averaged over all N treatments under B 0.5%, B 1% and B 2%, respectively, compared to no amendment. There was no interaction effect between N fertilizer and biochar amendment on soil NO_3_^-^-N concentration.

#### Experiment 2

Addition of dolomite and biochar significantly affected (p<0.05) soil pH (Table 2). However, soil pH was not affected by soil moisture content and no other interaction showed significant effect on soil pH. Dolomite application significantly increased soil pH in both moisture contents. At 50% WFPS, a pH increase of 0.45 units was observed in soil treated with dolomite and of 0.56 units at 90% WFPS compared to the control soil. Biochar amendment significantly increased soil pH. The average increase in soil pH due to biochar amendment ranged from 0.03 to 0.08 units and 0.04 to 0.08 units at 50% and 90% WFPS, respectively, compared to no biochar amendment soil.

**Table 2:** Soil total N, total C and C/N ratio and C/IN ratio after 60 days of incubation (Experiment 2). Numbers in the table represent means ± standard deviation. Data analyzed by three-way ANOVA with the factors of Dolomite (D), Biochar (B) and WFPS (%). (D0—No Dolomite, D1—Dolomite, B0—No biochar, RH—Rice husk, SD—Sawdust, BB—Bamboo).

WFPS	Treatment	Total N	Total C	C/N	C/IN	Soil pH
50%	D0-B0	6.5±0.46	69.0±5.0	10.6±0.14	88.4±8.2	3.38±0.01
	D0-RH	6.2±0.30	72.5±3.7	11.6±0.74	98.8±3.5	3.41±0.01
	D0-SD	6.8±0.48	84.2±5.8	12.4±0.58	115.2±13.9	3.46±0.01
	D0-BB	6.0±0.29	76.1±4.0	12.6±0.18	111.1±11.9	3.44±0.01
	D1-B0	6.1±1.16	64.2±13.5	10.5±0.21	78.5±15.0	3.83±0.12
	D1-RH	5.9±0.44	70.1±4.2	12.0±1.12	92.9±8.8	3.90±0.04
	D1-SD	6.4±0.55	88.6±5.8	13.9±1.62	116.3±5.6	3.87±0.08
	D1-BB	6.1±0.41	79.0±10.6	12.9±0.85	104.7±12.7	4.04±0.04
90%	D0-B0	6.0±0.34	63.7±2.9	10.7±0.19	99.5±21.1	3.40±0.08
	D0-RH	6.1±0.67	70.1±5.3	11.5±0.52	104.7±7.3	3.44±0.05
	D0-SD	6.0±0.21	70.9±1.3	11.9±0.22	119.8±9.6	3.48±0.06
	D0-BB	6.3±0.62	85.0±9.4	13.4±0.30	169.1±42.9	3.47±0.02
	D1-B0	6.1±0.93	65.4±9.1	10.7±0.29	103.1±5.5	3.96±0.15
	D1-RH	6.5±0.62	74.1±8.6	11.4±0.47	127.9±19.2	4.03±0.04
	D1-SD	6.7±0.67	84.1±8.6	12.6±0.72	142.3±5.4	3.99±0.06
	D1-BB	5.5±0.97	75.3±17.1	13.5±0.85	123.4±17.5	4.10±0.12
Analysis of Variance						
Dolomite (D)		ns	ns	ns	ns	**
Biochar (B)		ns	**	**	**	*
WFPS (%)		ns	ns	ns	**	ns
D x B		ns	ns	ns	ns	*
D x WFPS		ns	ns	ns	ns	ns
B x WFPS		ns	ns	ns	ns	ns
D x B x WFPS		ns	ns	ns	ns	ns

*P<0.05

**P<0.01, ns = not significance at 0.05 level

Soil total N content was not affected by soil moisture content, dolomite or biochar amendment after 60 days of incubation (Table 2). Biochar amendment significantly (p<0.01) affected soil total C content. The mean soil total C content for the control, RH, SD, and BB biochar were 65.6, 71.7, 81.9, and 78.9 g kg^-1^ soil, respectively. Soil total C content was not affected by either soil moisture or dolomite application and no interaction effect was observed among the treatments. Soil C/N ratio was only affected by biochar amendment. Soil C/IN ratio was significantly (p<0.01) affected by soil moisture and biochar amendment after 60 days of incubation. High soil C/N ratios and C/IN ratios were observed in all biochar treatments compared to no biochar. An increase in C/IN ratio at 90% WFPS was observed compared to 50% WFPS. There was no interaction effect on soil C/N and C/IN ratio among the treatments at the end of incubation.

**Fig 1:**
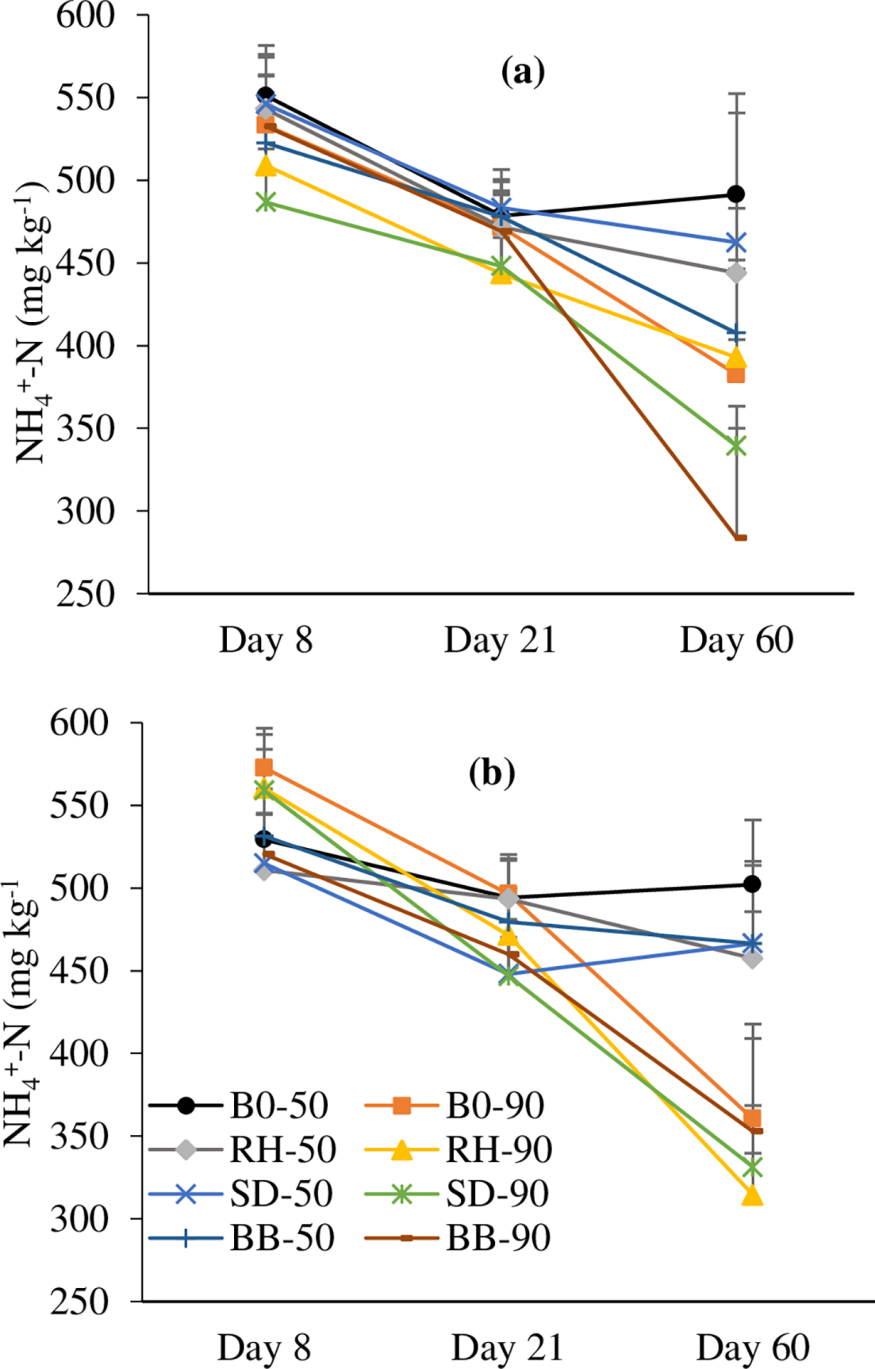
Soil NH_4_^+^-N concentration during 60-day incubation period; (a) No dolomite and (b) Dolomite application. Error bars indicate standard deviation. B0—No biochar, RH—Rice husk, SD—Saw dust, BB—Bamboo, 50–50% WFPS, 90–90% WFPS.

After 8 days of incubation, soil NH_4_^+^-N concentration was not affect by dolomite, biochar or soil moisture content (Fig 1 & S2 Table). After 21 and 60 days of incubation, soil NH_4_^+^-N concentration was also not affected by either dolomite and biochar amendment. However, soil NH_4_^+^-N concentration was significantly (p<0.05) affected by soil moisture content. High NH_4_^+^-N content was observed at 50% WFPS compared to 90% WFPS. There was no interaction effect on soil NH_4_^+^-N concentration among the treatments.

**Fig 2:**
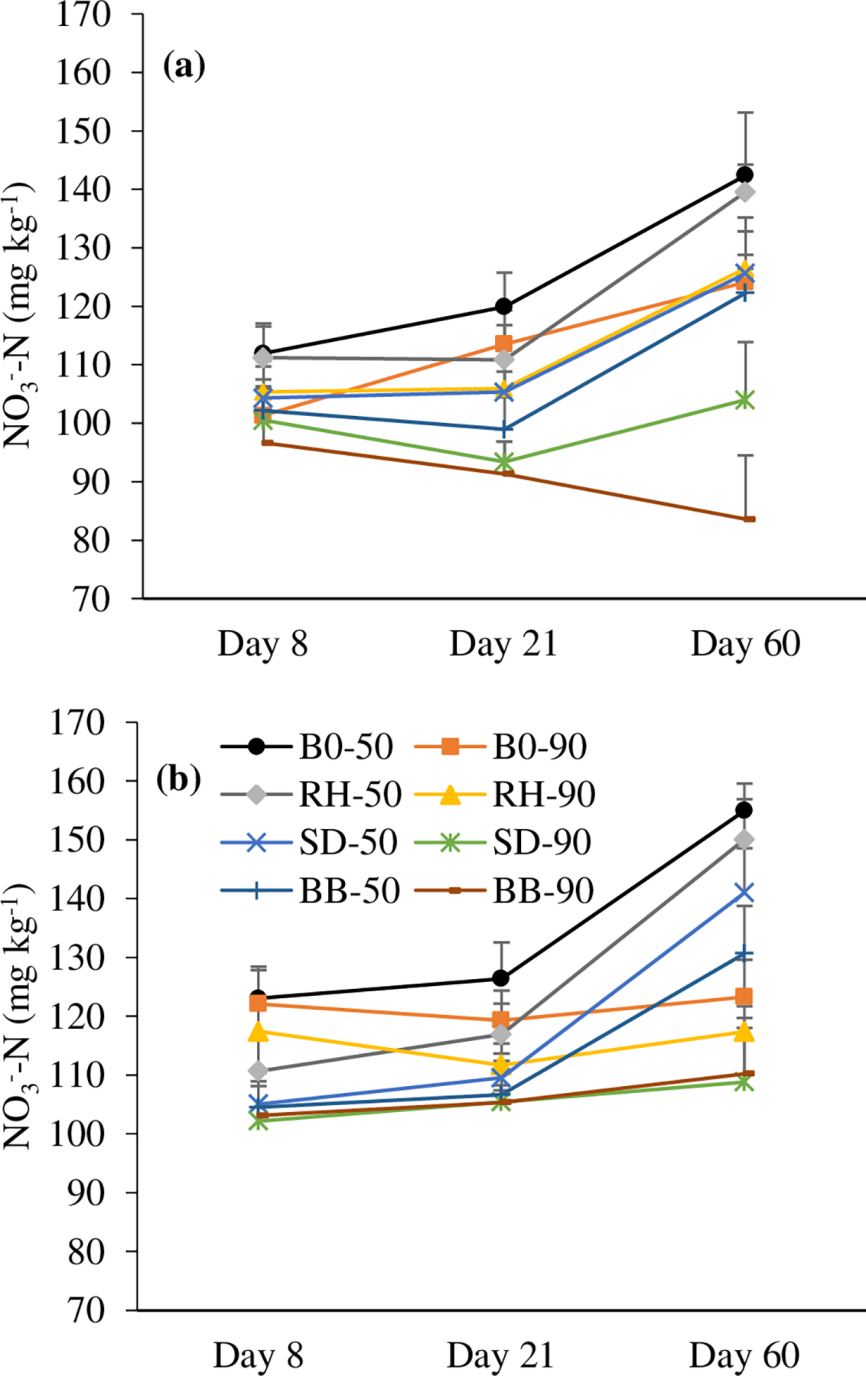
Soil NO_3_^-^-N concentration during 60-day incubation period; (a) No dolomite and (b) Dolomite application. Error bars indicate standard deviation. B0—No biochar, RH—Rice husk, SD—Saw dust, BB–Bamboo, 50–50% WFPS, 90–90% WFPS.

Soil NO_3_^-^-N concentration was significantly affected by dolomite (p<0.01) and biochar amendment (p<0.01) after 8 days of incubation (Fig 2 & S2 Table). Significant (p<0.05) interaction effect was also observed between treatments. There were significant effects of dolomite (p<0.05), biochar (p<0.01) and soil moisture (p<0.001) on soil NO_3_^-^-N concentration after 21 and 60 days of incubation. The mean NO_3_^-^-N concentration was 109.7 and 132.8 mg kg^-1^ soil under dolomite application and 103.7 and 122.0 mg kg^-1^ soil in the control after 21 and 60 days of incubation, respectively. Conversely, biochar amendment significantly decreased soil NO_3_^-^-N content. After 60 days of incubation, the mean soil NO_3_^-^-N content for the control, RH, SD, and BB biochar were 138.7, 134.3, 121.1, and 115.4 mg kg^-1^ soil, respectively. An increase in soil NO_3_^-^-N content at 50% WFPS (108 and 136.5 mg kg^-1^ soil) was observed compared to 90% WFPS (104 and 112.2 mg kg^-1^ soil after 21 and 60 days of incubation, respectively). There was no interaction effect on soil NO_3_^-^-N concentration among the treatments.

### N_2_O emissions

#### Experiment 1

**Fig 3:**
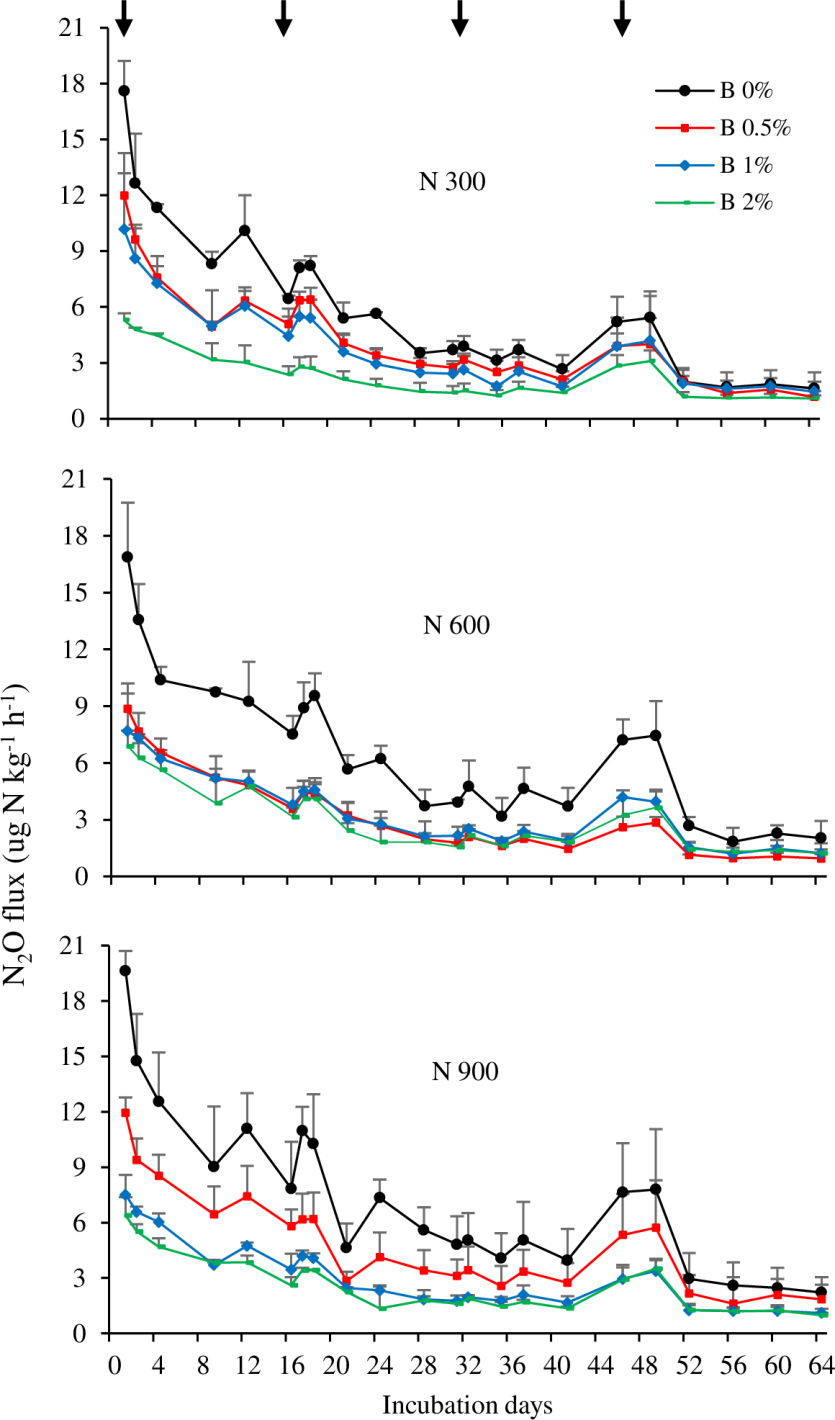
N_2_O flux from the soil treated with different rates of nitrogen and biochar (Experiment 1). Error bars indicate standard deviation. Arrows indicate the first, second, third and fourth split applications of N fertilizer.

The rate of N_2_O emission showed decreasing trend in all the N fertilizer treatments (Fig 3). The highest emission peak was observed at the beginning of experiment and other peaks appeared following the split applications of N fertilizer on 16, 32 and 46 days of incubation. The rate of N application significantly affected N_2_O emission. The lowest cumulative N_2_O emission was observed with N 300 (6.1 mg N_2_O-N kg^-1^ soil) (Table 3). When the N rate increased to 600 kg, only 0.8 units N_2_O-N increased was observed over N 300 (6.9 mg N_2_O-N kg^-1^ soil). The highest cumulative N_2_O emission was observed with the highest N rate of N 900 (7.7 mg N_2_O-N kg^-1^ soil).

**Table 3:** Cumulative N_2_O and CO_2_ emissions after 64 days of incubation. Data analyzed by two-way ANOVA with the factors of Nitrogen (N) and Biochar (B) (Experiment 1). Values are mean ± standard deviations.

Treatments	Cumulative N_2_O (mg N kg^-1^ soil)			Cumulative CO_2_ (mg C kg^-1^ soil)		
	N 300	N 600	N 900	N 300	N 600	N 900
B 0	6.1±0.8	6.9±1.1	7.7±1.5	2210.2±176	1947.1±237	2123.7±330
B 0.5	4.4±0.3	3.4±0.5	5.1±1.4	1955.8±206	1895.8±216	2194.9±266
B 1	4.1±0.2	3.6±0.6	3.2±0.4	2147.2±312	2051.9±200	2019.9±188
B 2	2.4±0.5	3.1±0.7	2.7±0.3	2299.8±271	2490.4±466	2274.5±180
Analysis of Variance						
N	*			ns		
B	**			**		
N x B	**			ns		

*P<0.05

**P<0.01, ns = not significance at 0.05 level

Bamboo biochar amendment significantly reduced N_2_O emissions from soils regardless of the quantity of N fertilizer used in this experiment (Fig 3 & Table 3). There was a clear tendency for the N_2_O flux to decrease as the rate of biochar application increased throughout the whole incubation period. At the end of incubation, the mean cumulative emissions for 0%, 0.5%, 1% and 2% biochar applications were 6.9, 4.3, 3.6 and 2.7 mg N_2_O-N kg^-1^ soil, respectively. The rate of emission for 0.5%, 1% and 2% biochar significantly (P<0.01) declined by 38%, 48% and 61%, respectively, compared to 0% biochar. Based on overall difference between N treatments with and without biochar, the 2% biochar application reduced N_2_O emissions compared to 0% biochar by 60.7%, 55.1% and 64.9% from 300 N, 600 N and 900 N treated soils, respectively.

#### Experiment 2

**Fig 4:**
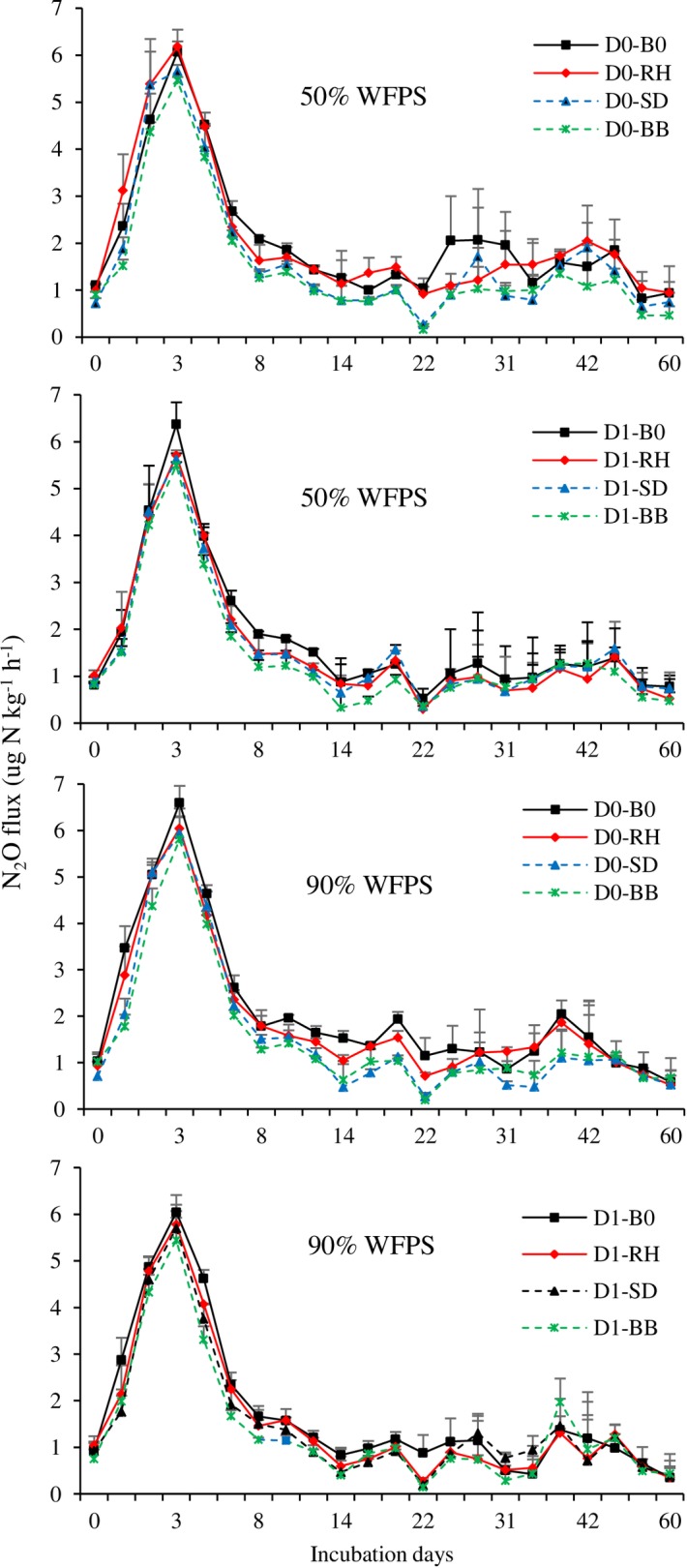
Emission of N_2_O from the soil treated with dolomite and biochar under 50% and 90% WFPS (Experiment 2). Error bars indicate standard deviation. (D0—No dolomite, D1—dolomite, B0—No biochar, RH—Rice husk, SD—Sawdust, BB—Bamboo).

N_2_O emissions dramatically increased following the application of N fertilizer, and the greatest N_2_O flux occurred between days 0–3 of incubation and then sharply decreased from day 3 to day 8 of incubation, after which N_2_O emissions remained steady with only small fluxes (Fig 4). There were significant effects of dolomite (p<0.001) and biochar (p<0.001) on N_2_O emissions. The interaction of dolomite and biochar treatments had a significant (p<0.01) effect on soil N_2_O emission, whereas no other interaction showed a significant effect. Higher magnitudes of N_2_O emissions were observed at 50% WFPS compared to 90% WFPS (Table 4). The mean cumulative N_2_O emission was 2.51 mg N kg^-1^ at 50% WFPS and 2.42 mg N kg^-1^ at 90% WFPS.

**Table 4:** Cumulative N_2_O and CO_2_ emissions after 60 days of incubation. Data analyzed by three-way ANOVA with the factors of dolomite (D), biochar (B) and water filled pore space (WFPS) (Experiment 2). Error bars indicate standard deviation. D0—No dolomite, D1—dolomite, B0—No biochar, RH—Rice husk, SD—Sawdust, BB–Bamboo.

Treatments	Cumulative N_2_O (mg N kg-1 soil)		Cumulative CO_2_ (mg C kg-1 soil)	
	50% WFPS	90% WFPS	50% WFPS	90% WFPS
L0-B0	3.03±0.41	3.04±0.25	3050.7±316.1	4188.8±218.6
L0-RH	3.02±0.31	2.75±0.14	3975.6±319.1	4488.3±530.2
L0-SD	2.50±0.23	2.33±0.19	3476.0±168.6	4381.7±586.0
L0-BB	2.19±0.10	2.24±0.18	3446.9±562.4	4225.9±825.4
L1-B0	2.61±0.11	2.52±0.26	3308.7±820.1	4287.6±308.4
L1-RH	2.32±0.01	2.26±0.21	3692.9±316.7	4919.2±599.8
L1-SD	2.33±0.26	2.19±0.25	3278.3±493.1	4707.7±474.1
L1-BB	2.06±0.12	2.03±0.12	3548.1±129.5	4362.6±129.5
Analysis of Variance				
Dolomite (D)	**		ns	
Biochar (B)	**		*	
WFPS (%)	ns		**	
D x B	**		ns	
D x WFPS	ns		ns	
B x WFPS	ns		ns	
D x B x WFPS	ns		ns	

*P<0.05

**P<0.01, ns = not significance at 0.05 level

Addition of dolomite significantly (p<0.001) reduced N_2_O emissions under both soil moisture conditions (Fig 4 & Table 4). The mean cumulative N_2_O emission was 2.64 mg N kg^-1^ without dolomite and 2.29 mg N kg^-1^ with dolomite application. The rate of emission from dolomite treated soil decreased by 13.3% compared to untreated. Biochar amendment significantly (p<0.001) reduced N_2_O emissions but the magnitude of reduction differed among the different biochar types. At the end of incubation, the cumulative N_2_O emission for the control, RH, SD, and BB biochar were 2.8, 2.6, 2.3, and 2.1 mg N kg^-1^ soil, respectively. The rate of emissions for RH, SD, and BB biochar decreased by 4.6%, 20.5%, and 26.7%, respectively, compared to no biochar amendment. Because of significant (p<0.01) interaction effect of dolomite and biochar, a high percentage of reduction in N_2_O emission under dolomite application was observed with RH (24.4%), SD (25.4%), and BB (32.7%) compared to the control soil.

### CO_2_ emissions

#### Experiment 1

**Fig 5:**
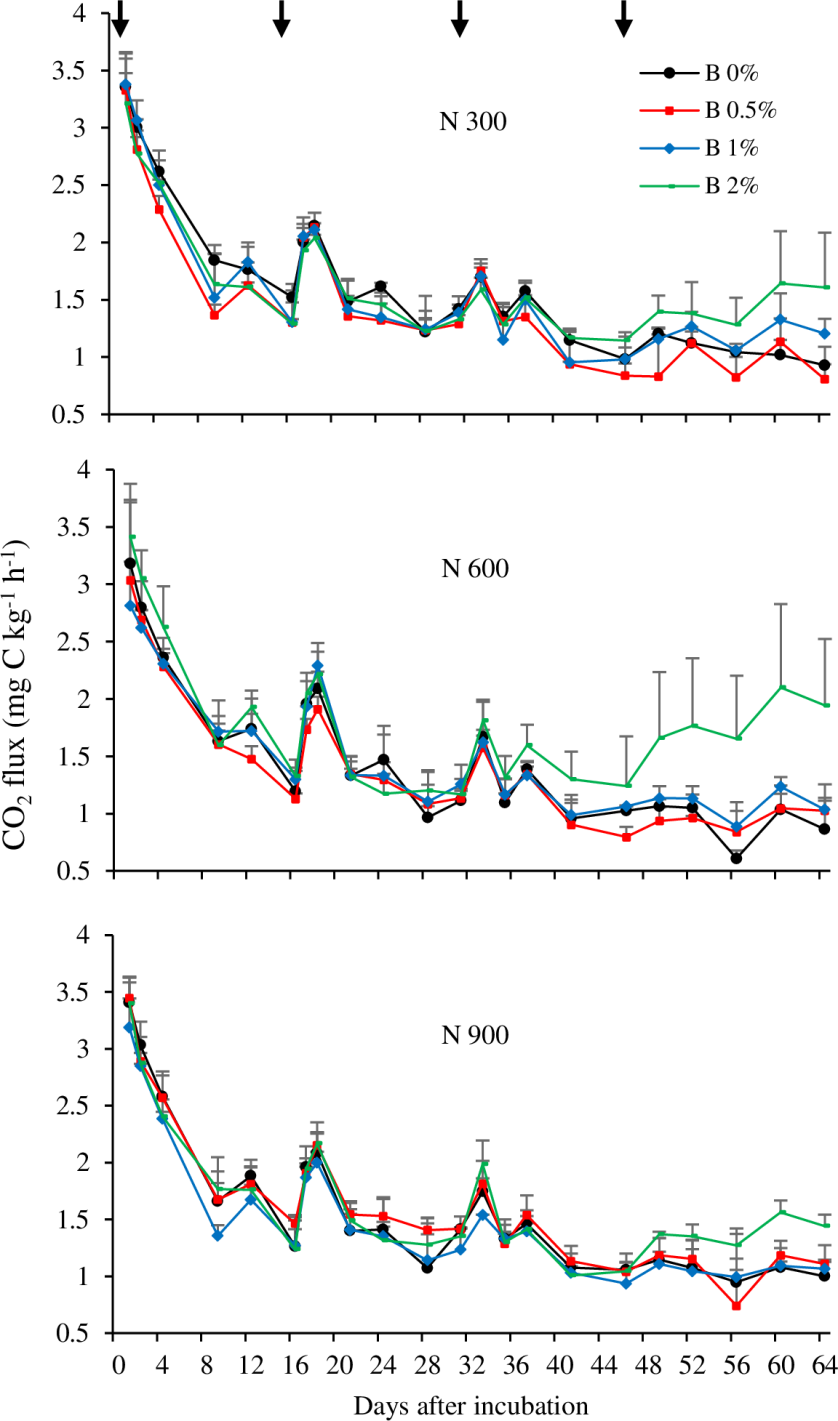
CO_2_ flux from the soil treated with different rates of nitrogen and biochar (Experiment 1). Error bars indicate standard deviation. Arrows indicate the first, second, third and fourth split applications of N fertilizer.

There was a gradual decline in CO_2_ flux over time in all the N fertilizer treatments, with the highest CO_2_ emission peak occurring within the first day of incubation (Fig 5). Other medium to low CO_2_ emission peaks were observed following split application of N fertilizer on 16, 32 and 46 days of incubation. No significant difference in cumulative CO_2_ emission was observed among the N treatments (Table 3). The mean cumulative CO_2_ emissions from N 300, N 600 and N 900 at the end of the incubation period were 2210.2, 1947.1 and 2123.7 mg CO_2_-C kg^-1^ soil, respectively.

In biochar amendments, CO_2_ emission also showed decreasing trend toward the end of the incubation period except 2% biochar which increased again after 46 days of incubation (Fig 5). High emission peak was observed in the first day of incubation. Other CO_2_ emission peaks appeared after split applications of N fertilizer on 16, 32 and 46 days of incubation. But only significant (P<0.05) higher CO_2_ emissions were observed after fourth time split application of N fertilizer in all soils treated with 2% biochar. Bamboo biochar application significantly affected (P<0.05) cumulative CO_2_ emission (Table 3). Treatment B 2% showed the highest cumulative CO_2_ emission while significant differences between the control, B 0.5% and B 1% were not found.

#### Experiment 2

**Fig 6:**
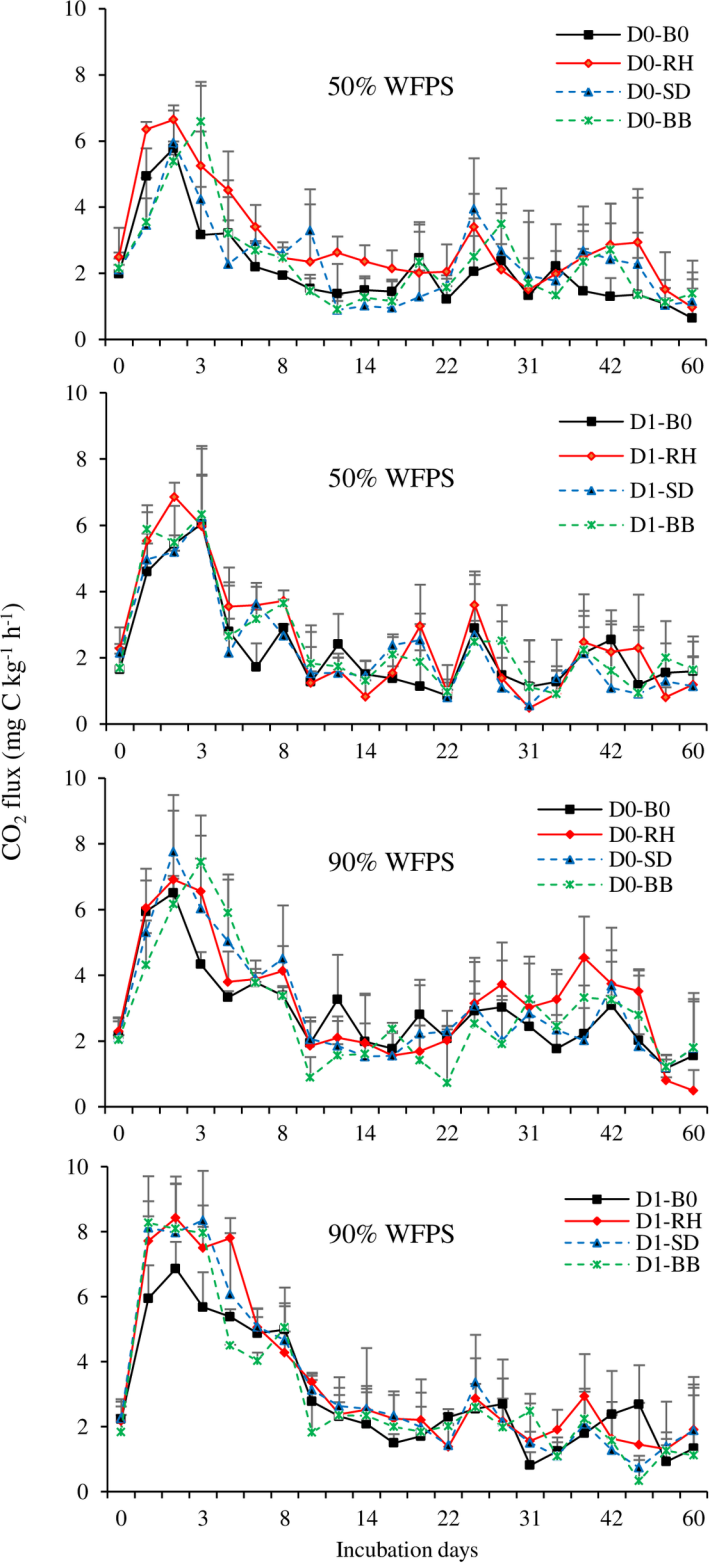
Emission of CO_2_ from the soil treated with dolomite and biochar under 50% and 90% WFPS (Experiment 2). Error bars indicate standard deviation. (D0- No dolomite, D1 –dolomite, B0 –No biochar, RH—Rice husk, SD–Sawdust, BB–Bamboo).

A sharp increase in CO_2_ emission was observed following the application of N fertilizer with the peak emission on day 3 of incubation and then decreased on following days (Fig 6). Subsequently, CO_2_ emissions remained steady with small fluxes in the later incubation period. There were significant effects of biochar (p<0.05) and soil moisture (p<0.001) on CO_2_ emission (Table 4). However, soil CO_2_ emission was not significantly affected by dolomite application and there was no interaction effect among the treatments. Relatively high CO_2_ emission after dolomite (4013 mg C kg^-1^ soil) application was observed compared to the control (3904 mg C kg^-1^ soil). Higher magnitudes of CO_2_ emissions were observed at 90% WFPS compared to 50% WFPS. The mean cumulative CO_2_ emission was 3472 mg C kg^-1^ soil at 50% WFPS and 4445 mg C kg^-1^ soil at 90% WFPS. Relative to 50% WFPS, mean cumulative CO_2_ emission was 27.8% higher at 90% WFPS. Biochar amendment significantly (p<0.05) increased CO_2_ emissions under both soil moisture conditions. At the end of incubation, the mean cumulative CO_2_ emission for the control, RH, SD, and BB biochar were 3709, 4269, 3960, and 3896 mg C kg^-1^ soil respectively. The rate of emission for RH, SD, and BB biochar significantly (p<0.05) increased by 15%, 11%, and 5% respectively, compared to no biochar amendment. Among the different biochar types, RH showed the highest cumulative CO_2_ emission while significant differences between SD and BB biochar were not found.

### Correlation of cumulative gas emissions and soil properties

**Fig 7:**
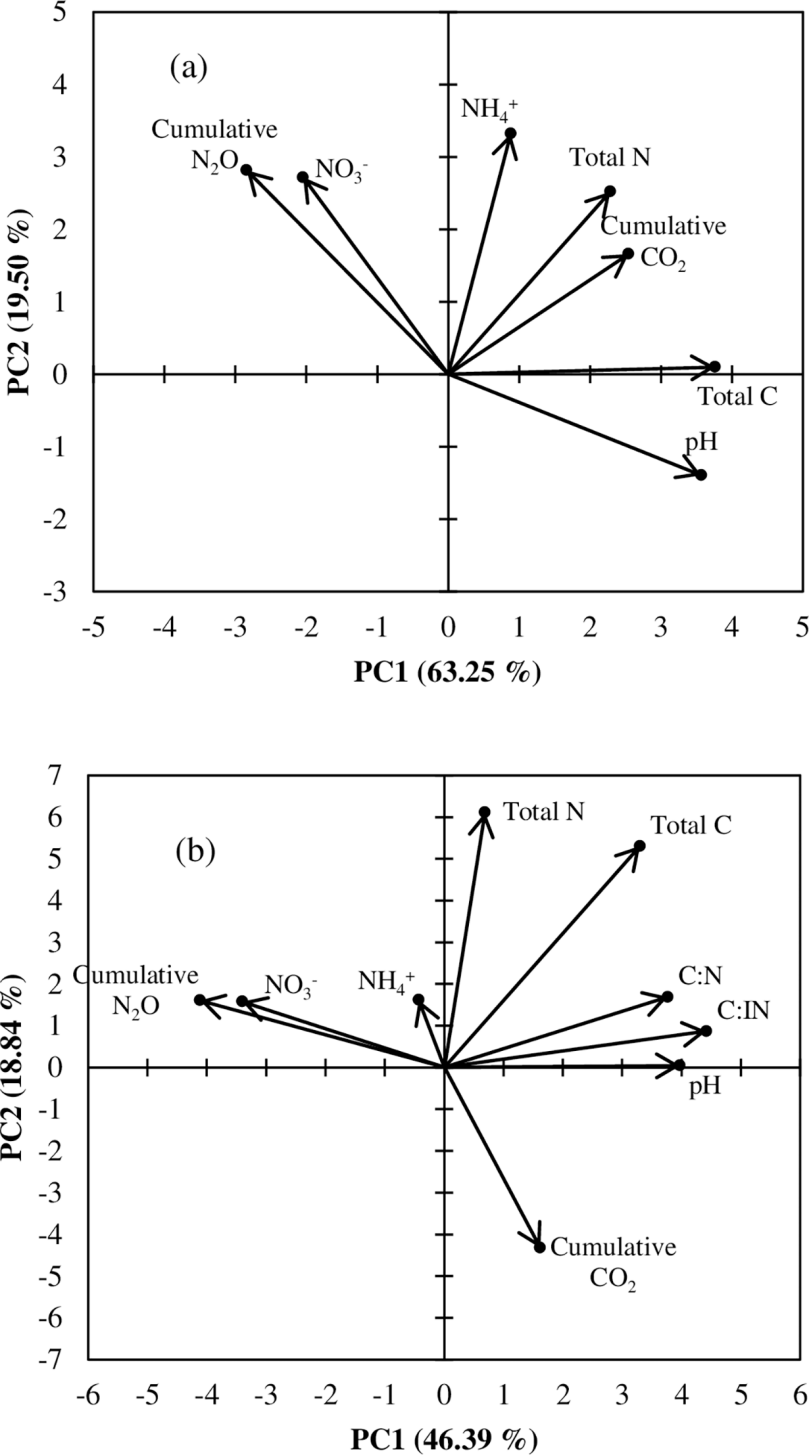
Correlation triplot based on a principal component analysis (PCA) using cumulative emissions and soil properties; a PC2 versus PC1 loadings for the included variables: (a) Experiment 1 and (b) Experiment 2. Arrows indicate vectors for both gas emissions and soil properties, with longer arrows indicating higher influence for those parameters. The direction of an arrow indicates the steepest increase in the variable and the length indicates the strength relative to the other variables.

**Fig 8:**
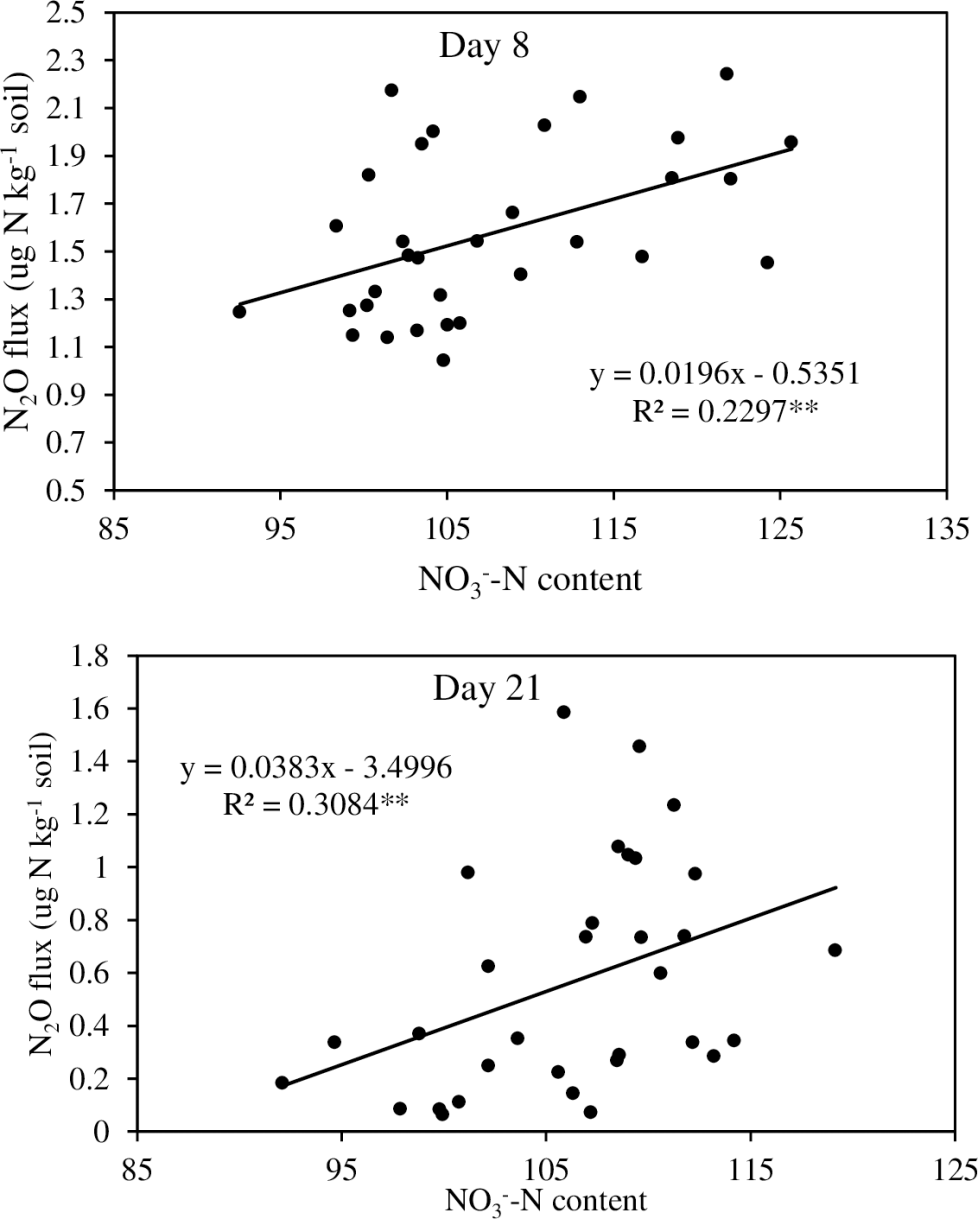
Correlation between N_2_O emission flux and soil NO3—N concentration on day 8 and 21 of incubation (Experiment 2). **P<0.01.

A principal component analysis (PCA) was performed to explore the soil properties that related with cumulative N_2_O and CO_2_ emissions (Fig 7). A vector represented each variable, and the length of each vector indicated the strength of its contribution. From the perpendicular projection of each sample to its respective vector, the relative importance of each variable can be estimated. The two main axes (PC1 and PC2) indicated the total variance of the data explained in the PCA. In experiment 1, the first principal component explained about 63% of the observed variation, while the second component accounted for 19% of the observed variation (Fig 7A). The decrease in soil N_2_O emissions was highly related with soil NO_3_^-^-N concentrations and inversely related with soil pH and total C content. In experiment 2, the first principal component explained about 36% of the observed variation, while the second component accounted for 22% (Fig 7B). The decrease in soil N_2_O emissions was highly related also with soil NO_3_^-^-N concentrations and inversely related with soil pH, C:N and C:IN ratios. According to correlation analysis, soil NO_3_^-^-N contents on day 8 and 21 of incubation were significantly related with their respective N_2_O fluxes (Fig 8).

## Discussion

### N_2_O emissions

N_2_O emissions were significantly lower in the dolomite-treated soil than in the untreated soil (Fig 4 & Table 4). Decreased N_2_O emissions have also been reported in lime-treated soils in previous studies [13, 14]. The application of lime to acidic soils reduced the N_2_O emissions, which might have occurred through an increase in soil pH [28]. This result was further supported by correlation analysis, which revealed that N_2_O emission was negative related to soil pH (Fig 7). The change in pH of dolomite-treated soil increased the N_2_O-reductase activity and consequently reduced the N_2_O emissions [28]. A low soil pH inhibited the activity of this enzyme and restrained the production of N_2_O reductase [29]. However, Baggs et al. [11] and Higgins et al. [30] observed that lime-treated soil produced larger amounts of N_2_O when compared to the un-limed soil. Such contradictory results could be because of different soil properties, microbial populations, and communities that responded differently to the manipulation of soil pH [31]. The results of the present study demonstrate that an increase in soil pH following dolomite application decreased N_2_O emissions in the acidic tea field soil.

Biochar addition has been reported to have positive, negative, or negligible effect on soil N_2_O emissions [20, 32, 33, 34]. This apparent inconsistency might be because of the differences in the biochar types and properties of the soils used. In experiment 1, N_2_O emission from acidic tea field soil was observed to be significantly influenced by N fertilizer and biochar amendment (Fig 3 & Table 3). Regardless of the quantity of N fertilizer used, the bamboo biochar significantly reduced the N_2_O emission from the tea field soil. In experiment 2, all the biochar amendments significantly reduced the N_2_O emission compared to the emission obtained in the control (no amendment); however, the magnitude of reduction differed among the different biochar types (Fig 4 & Table 4). The reduction in N_2_O emission upon biochar amendment has also been reported by Ameloot et al. [35] and Cayuela et al. [34]. All the biochar amendments increased the soil pH compared to the pH of the control soil (Tables 1 & 2). The results of principal component analysis showed that N_2_O emission was negatively related to the soil pH (Fig 7). Therefore, biochar application might be responsible for this reduction through an increase in pH, with low pH preventing the assembly of functional N_2_O reductase (N_2_OR), the enzyme that reduces N_2_O to N_2_ during the process of denitrification [36, 37]. Because of the retention of water in their fine pores and its capacity to create local alkaline conditions, biochar particles might also act as hotspots for complete denitrification [35]. It is, thus, likely that in these microsites the intermediary product, N_2_O, might be completely reduced to N_2_. However, the increase in soil pH under biochar amendment was relatively small, ranging from 0.07 to 0.09 units in experiment 1 (Table 1) and from 0.03 to 0.08 units in experiment 2 (Table 2) over that in the control. Therefore, the reduction in N_2_O emission upon biochar amendment might also be influenced by other factors.

Downie et al. [38] reported that increased soil aeration upon biochar addition might also be responsible for reducing the N_2_O emissions. The addition of porous biochar might increase soil aeration, which suppresses denitrification [17]. Reduced NO_3_^-^ availability as a result of microbial N-immobilization during microbial consumption of N-depleted volatile biochar compounds might be another possible mechanism for reduced N_2_O emission upon biochar amendment [35].

In experiment 2, biochar amendment increased the soil C/N and C/IN ratios, which could be the key parameters affecting the utilization of soil N. A strong negative relation between N_2_O emission and soil C/N and C/IN ratios suggested that soil C/N and C/IN ratios were also the influencing factors affecting N_2_O emission (Fig 7). Feng et al. [39] stated that when the soil C/N ratio increases, the N demand of microbes increases above the N availability and N becomes a limiting factor relative to C for nitrification or denitrification and, thus, N_2_O emission becomes relatively low. Ernfors et al. [40] also reported that soil C/N ratio was negatively correlated with N_2_O emission, although Feng et al. [39] found no significant correlation. Previous studies indicated that as the relative C content increases in soil, a higher proportion of ammonium is immobilized (or assimilated) by microbes instead of being nitrified (or mineralized), leading to a decrease in soil inorganic N and suppression of N_2_O emission [41]. They also discussed the possibility that not all the N in soil and biochar was directly associated with N_2_O emission and the nitrogen involved was mainly inorganic N rather than organic N. Feng et al. [39], therefore, suggested that biochar-induced change in soil C/IN was probably the dominant factor for N_2_O emission under biochar amendment. Different types of biochars gave different magnitudes of N_2_O reduction compared to that in the control (Table 4). The N_2_O emission value was lowest in the case of Bamboo biochar (BB) amendment followed by that in SD and RH biochar amendments in experiment 2. Cayuela et al. [42] also reported that biochars made from bamboo and oak significantly reduced the N_2_O emissions. A high relation between N_2_O emission and NO_3_^-^-N (Figs 7 & 8) suggested that low soil NO_3_^-^ concentration in BB at the end of incubation resulted in significantly lower N_2_O emission.

In this study, consistently lower soil NO_3_^-^-N concentrations observed on day 8, 21, and 60 of incubation (Experiment 2) in the biochar-amended soils relative to that in the control (Fig 2) support the hypothesis that biochar reduces N_2_O emissions by reducing the NO_3_^-^-N availability. A similar result was also observed by Cayuela et al. [34]. A strong correlation was observed between soil NO_3_^-^-N concentration and N_2_O emissions (Fig 8). A reduction in NO_3_^-^ availability would indeed decrease the total N that is denitrified and it would favor the last step of denitrification [43]. The reduced N_2_O emission upon biochar amendment might partly be explained by the reduction in the availability of soil NO_3_^-^-N, which might have reduced the soil inorganic-N pool for N_2_O production in this experiment. The reduction in N_2_O emissions in this short-term incubation experiment might be due to one or more of the biochar-induced mechanisms reported in the literature and collated by Ameloot et al. [35], namely increased pH, enhanced soil aeration, surface adsorption of the gas, changes in microbial processes, and reduced soil inorganic-N pool for N_2_O production. Although biochar has the potential to mitigate N_2_O emissions from acidic tea field soils, it can only be applied either in the fertilizer application trenches or in rows/inter-rows under field conditions and complete mixing of biochar and tea soil will be limited in the tea landscape.

### CO_2_ emissions

An initial sharp increase in CO_2_ emissions after ammonium fertilization in all treatments was observed during the first 3 days of incubation followed by a rapid decrease which became more gradual (Figs 5 & 6). This pattern of emission was due to rapid mineralization of the readily decomposable soil organic carbon [44]. Our result for CO_2_ production was in close agreement with the study of Kong et al. [45], which also observed the highest CO_2_ production during the initial phase of the incubation, and then it decreased with incubation time.

Soil CO_2_ emissions have been reported to increase [22], decrease [23] and remain unchanged [24] with biochar amendment. In this study, cumulative CO_2_ emission was significantly higher under different biochar amendments compared to the control (Tables 3 & 4). Possible reasons for increasing CO_2_ production were mineralization of labile C added with the biochar [23, 46], enhanced mineralization, or priming of the soil organic matter due to stimulation caused by the addition of a labile C source [23], and increased soil surface area due to pore structures which promotes microbial activity [47]. Another reason for increasing CO_2_ production under biochar amendment is assumed to be an accelerated rate of soil organic matter mineralization caused by increased soil aeration due to the lower bulk density of the biochar-amended soil, which resulted in higher aerobic microbial activity and enhanced microbial colonization, causing accelerated decomposition of organic compounds [48]. In this study, increased in soil CO_2_ emissions under biochar amendment was influenced by TC, C/IN and NO_3_^-^-N content since these factors are highly related with CO_2_ emission (Fig 7).

Lime is considered to improve soil conditions by increasing soil pH and thus increasing microbial respiration and loss of soil organic carbon as CO_2_ [49] but reported results on effects of lime on CO_2_ emissions are highly diverse [50, 51, 52]. In experiment 2, although higher CO_2_ emission was observed under dolomite application compared to without dolomite, the result was not statistically different. Soil pH played an important role in CO_2_ emissions through its influence on microbial decomposition of soil organic matter [10]. Although dolomite application significantly increased soil pH, the low correlation between soil pH and CO_2_ emission suggested that pH had no effect on soil CO_2_ emission (Fig 7). Another reason might be that dolomite was not readily soluble in water and there was no CO_2_ release from dolomite [52].

The mineralization of organic matter is mainly controlled by the biological activities in soil and is affected by soil temperature and moisture [53]. Many studies have showed that CO_2_ emission increases as the water content increases in peat soil [53], paddy soil [54], and forest soil [55]. However, CO_2_ emission decreases when soil water content exceeds 70% WHC [56]. Raich et al. [57] reported that near-anaerobic conditions could have inhibited microbial activities and reduced CO_2_ emission. In this study, regardless of biochar and dolomite application, the cumulative CO_2_ emission at 90% WFPS was significantly higher than that at 50% WFPS (Table 4). The most likely reason for this is the high porosity of Andosol soil, which tended to maintain aerobic conditions [58, 59]. Another possible reason is higher mineralization rates at high moisture contents, therefore, producing larger amounts of easily available organic C, which stimulates microbial activity to increase CO_2_ emission. Bolan et al. [9] discussed that soil moisture controls the mineralization of organic C, which is used as a substrate and an energy source by microbe for metabolism and activity.

## Conclusion

N_2_O emissions from acidic soil were consistently suppressed by bamboo biochar amendments of 0.5% and above in experiment 1. Changes in soil pH and magnitudes of soil mineral-N under biochar amendment was recognized as the driving factor for N_2_O emission change after application of bamboo biochar to acidic tea field soil. In experiment 2, the application of dolomite and biochar either individually or in combination reduced N_2_O emissions by 4.6% to 32.7% from acidic tea field soil. The reduction in N_2_O emission was affected by increase of soil pH, soil C/N and C/IN ratios and low NO_3_^-^-N concentration. Among the three different biochars, bamboo biochar produced by auto-thermal process showed the lowest N_2_O emission. However, addition of biochar to soil enhanced CO_2_ emission from acidic soil. The results suggest that the application of dolomite and biochar (either alone or combined) is a potential mitigation strategy for N_2_O emissions in acidic tea field soil. However, further research is needed to investigate the effects of dolomite and biochar on N_2_O emissions in acidic soil under field conditions.

## Supporting information

S1 Table Basic properties of different biochar. (DOCX)

S2 Table Analysis of variance for soil NH_4_^+^ and NO_3_^-^ concentration during 60-day incubation period. (DOCX)

S1 Fig Making biochar in an open burn kiln. (DOCX)
