# Supplementary material for: Effect of dolomite and biochar addition on N2O and CO2 emissions from acidic tea field soil
Source: PLoS One. 2018 Feb 2;13(2):e0192235. doi: 10.1371/journal.pone.0192235 (PMC5796709; doi:10.1371/journal.pone.0192235)
Supplement: S1 Table — (DOCX) [file pone.0192235.s001.docx]

S1 Table. Basic properties of different biochar

| Biochar | Surface area (BET) m^2^/g | pH (1:5 H_2_O) | Total C  (g kg^-1^) | Total N  (g kg^-1^) | C:N  ratio | NH_4_^+^-N  (mg kg^-1^), | NO_3_^-^-N  (mg kg^-1^) |
| --- | --- | --- | --- | --- | --- | --- | --- |
| Rice husk (RH) | 52.6 | 8.2 | 361.9 | 5.7 | 63.5 | nd*^a^* | nd |
| Sawdust (SD) | 25.2 | 8.7 | 372.4 | 3.3 | 112.5 | nd | nd |
| Bamboo (BB) | 89.3^β^ | 8.2 | 424.8 | 1.8 | 236 | 1.4 | 17.1 |

*^a^* below limit of detection,

^β^ Yoshizawa S. Biochar for carbon storage in the soil and for soil improvement. TANSO. 2015. 270, 232-240. (in Japanese with English abstract).
