# Supplementary material for: Effect of dolomite and biochar addition on N2O and CO2 emissions from acidic tea field soil
Source: PLoS One. 2018 Feb 2;13(2):e0192235. doi: 10.1371/journal.pone.0192235 (PMC5796709; doi:10.1371/journal.pone.0192235)
Supplement: S2 Table — (DOCX) [file pone.0192235.s002.docx]

S2 Table. Analysis of variance for soil NH_4_^+^ and NO_3_^-^ concentration during 60-day incubation period

|  | Day 8 | | Day 21 | | Day 60 | |
| --- | --- | --- | --- | --- | --- | --- |
|  | NH_4_^+^ | NO_3_^-^ | NH_4_^+^ | NO_3_^-^ | NH_4_^+^ | NO_3_^-^ |
| Dolomite (D) | ns | ** | ns | ** | ns | ** |
| Biochar (B) | ns | ** | ns | ** | ** | * |
| WFPS (%) | ns | ns | * | * | ** | ns |
| D x B | ns | * | ns | ns | ns | * |
| D x WFPS | ns | ns | ns | ns | ns | ns |
| B x WFPS | ns | ns | ns | ns | ns | ns |
| D x B x WFPS | ns | ns | ns | ns | ns | ns |

*P<0.05, **P<0.01, ns= not significance at 0.05 level
