# Supplementary figures and images for: Effect of dolomite and biochar addition on N2O and CO2 emissions from acidic tea field soil
Source: PLoS One. 2018 Feb 2;13(2):e0192235. doi: 10.1371/journal.pone.0192235 (PMC5796709; doi:10.1371/journal.pone.0192235)

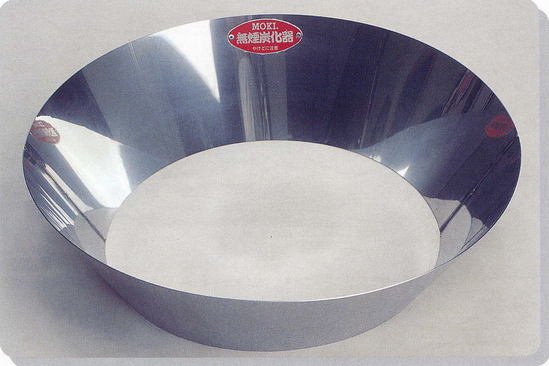

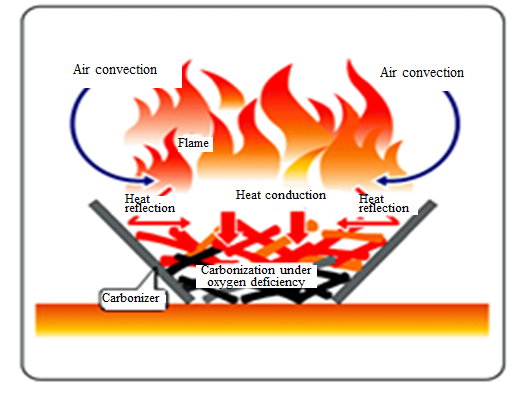


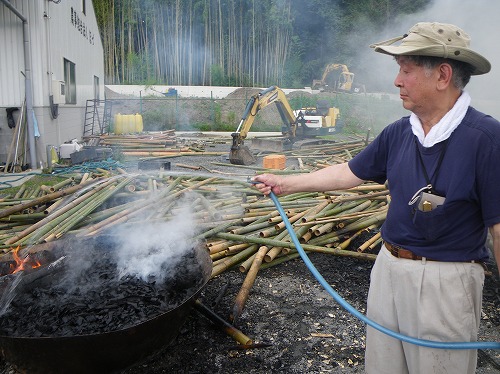

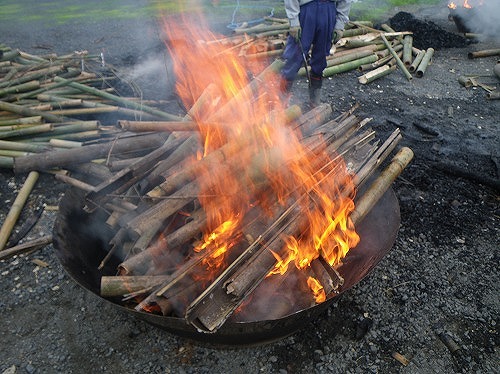


S1 Fig. Making biochar in an open burn kiln

Supplement: S1 Fig — (DOCX) [file pone.0192235.s003.docx]
